# Disturbance legacies increase and synchronize nutrient concentrations and bacterial productivity in coastal ecosystems

**DOI:** 10.1002/ecy.2988

**Published:** 2020-03-27

**Authors:** John S. Kominoski, Evelyn E. Gaiser, Edward Castañeda‐Moya, Stephen E. Davis, Shimelis B. Dessu, Paul Julian, Dong Yoon Lee, Luca Marazzi, Victor H. Rivera‐Monroy, Andres Sola, Ulrich Stingl, Sandro Stumpf, Donatto Surratt, Rafael Travieso, Tiffany G. Troxler

**Affiliations:** ^1^ Department of Biological Sciences & Institute of Environment/Southeast Environmental Research Center Florida International University Miami Florida 33199 USA; ^2^ Everglades Foundation Palmetto Bay Florida 33157 USA; ^3^ Department of Earth and Environment & Institute of Environment/Southeast Environmental Research Center Florida International University Miami Florida 33199 USA; ^4^ Whitney Laboratory for Marine Bioscience University of Florida Gainesville Florida 32611 USA; ^5^ Department of Oceanography and Coastal Sciences College of the Coast and the Environment Louisiana State University Baton Rouge Louisiana 70803 USA; ^6^ Institute of Food and Agricultural Sciences University of Florida Davie Florida 33314 USA; ^7^ National Park Service Boynton Beach Florida 33437 USA

**Keywords:** bacterioplankton, dissolved organic carbon, Florida Coastal Everglades, global climate change, long‐term ecological research, nitrogen, phosphorus, wetlands

## Abstract

Long‐term ecological research can resolve effects of disturbance on ecosystem dynamics by capturing the scale of disturbance and interactions with environmental changes. To quantify how disturbances interact with long‐term directional changes (sea‐level rise, freshwater restoration), we studied 17 yr of monthly dissolved organic carbon (DOC), total nitrogen (TN), and phosphorus (TP) concentrations and bacterioplankton productivity across freshwater‐to‐marine estuary gradients exposed to multiple disturbance events (e.g., droughts, fire, hurricanes, and low‐temperature anomalies) and long‐term increases in water levels. By studying two neighboring drainages that differ in hydrologic connectivity, we additionally tested how disturbance legacies are shaped by hydrologic connectivity. We predicted that disturbance events would interact with long‐term increases in water levels in freshwater and marine ecosystems to increase spatiotemporal similarity (i.e., synchrony) of organic matter, nutrients, and microbial activities. Wetlands along the larger, deeper, and tidally influenced Shark River Slough (SRS) drainage had higher and more variable DOC, TN, and TP concentrations than wetlands along the smaller, shallower, tidally restricted Taylor River Slough/Panhandle (TS/Ph) drainage. Along SRS, DOC concentrations declined with proximity to coast, and increased in magnitude and variability following drought and flooding in 2015 and a hurricane in 2017. Along TS/Ph, DOC concentrations varied by site (higher in marine than freshwater wetlands) but not year. In both drainages, increases in TN from upstream freshwater marshes occurred following fire in 2008 and droughts in 2010 and 2015, whereas downstream increases in TP occurred with coastal storm surge from hurricanes in 2005 and 2017. Decreases in DOC:TN and DOC:TP were explained by increased TN and TP. Increases in bacterioplankton productivity occurred throughout both drainages following low‐temperature events (2010 and 2011) and a hurricane (2017). Long‐term TN and TP concentrations and bacterioplankton productivity were correlated (*r* > 0.5) across a range of sampling distances (1–50 km), indicating spatiotemporal synchrony. DOC concentrations were not synchronized across space or time. Our study advances disturbance ecology theory by illustrating how disturbance events interact with long‐term environmental changes and hydrologic connectivity to determine the magnitude and extent of disturbance legacies. Understanding disturbance legacies will enhance prediction and enable more effective management of rapidly changing ecosystems.

## Introduction

Ecosystems are commonly exposed to disturbances yet understanding the spatial and temporal dynamics of disturbance events and consequences remains a fundamental challenge to the field of ecology (Peters et al. 2011, Grimm et al. 2017). Disturbances are considered relatively discrete events that alter the structure and function of ecosystems (Pickett and White 1985), but long‐term ecological research has identified how spatial and temporal trends and their drivers can emerge through the processes of disturbance events, effects, and recovery (Peters et al. 2011, Grimm et al. 2017, Gaiser et al. 2020). Ecological theory predicts that ecosystems are maintained and enhanced through regular, pulsed disturbances that release and redistribute organic matter and nutrients (Junk et al. 1989, Odum et al. 1995, Polis et al. 1997, Yang et al. 2008, 2010). Climate and hydrologic disturbances synchronize the structure and phenology of populations and communities (Yang and Rudolf 2010, Ruhí et al. 2018). However, how legacies of multiple disturbances occurring at different scales interact to affect ecosystem functioning is not well understood. Specifically, disturbance ecology theory needs to advance understanding of how (1) disturbances interact synergistically or antagonistically over time and space, and (2) disturbances interact with other drivers of environmental change (Turner 2010).

Long‐term ecological research offers us the opportunity to observe how disturbance events interact with long‐term drivers of environmental change to influence patterns and processes of ecosystems. Coastal ecosystems are highly altered by local to global‐scale drivers operating at different spatiotemporal scales. For example, changes in land‐use/land‐cover, accelerating rates of sea‐level rise and saltwater intrusion, climate‐driven changes in precipitation and temperature, and increasing intensity and frequency of storms are all drivers that act at multiple scales to influence coastal ecosystems (Nicholls and Cazenave 2010, Farfan et al. 2014, Pachauri et al. 2014 , Herbert et al. 2015, Osland et al. 2016, Tully et al. 2019). As coastal ecosystems are arguably among the most disturbed and disturbance‐adapted ecosystems worldwide (Odum et al. 1995), it is important to be able to parse the effects of both long‐term environmental changes and disturbance events in terms of their impacts on structural and functional responses.

Disturbance events, such as hurricanes and temperature anomalies, can result in pulsed releases of organic matter and nutrients influencing long‐term water quality dynamics in coastal wetlands (Davis et al. 2018). For example, between 2001 and 2017, multiple disturbance events impacted the Florida Coastal Everglades (see Davis et al. 2019). In the Everglades, major hurricanes made landfall in 2005 (Hurricane Wilma) and 2017 (Hurricane Irma) on the southwest coast of Florida as Category 3 storms with deposition of marine‐derived, P‐rich carbonate sediments documented in coastal mangrove forests (Castañeda‐Moya et al. 2010, Castañeda‐Moya et al. 2020). A major fire in 2008 occurred in upstream freshwater marshes of both drainages, followed by extensive drought in 2010 and 2015 (Davis et al. 2018). Fire is known to release P and increase N‐fixation in some ecosystems (Schafer and Mack 2010, Butler et al. 2018). Two record cold events affected all of South Florida in 2010 and 2011, leading to widespread fish mortality in freshwater and brackish marshes and defoliation and mortality of mangroves (Boucek and Rehage 2014, Boucek et al. 2016, Danielson et al. 2017, Davis et al. 2018), releasing autochthonous carbon and nutrient subsidies. Different disturbances likely have legacies that affect biogeochemistry in different ways (Peters et al. 2011, Grimm et al. 2017). Disturbance impacts on nutrient availability, carbon transport, and fate (use by microbes) may also depend on coastal drainage characteristics such as drainage area, flow, and sources of organic matter and nutrients. Quantifying relationships between disturbance events and their legacies on long‐term changes in biogeochemistry is important for predicting how disturbance legacies may interact with long‐term environmental changes to enhance synchrony in more than in less hydrologically connected ecosystems.

Hydrologic‐ and temperature‐driven environmental changes and disturbance events can impact the sources and timing of delivery of organic matter and nutrients in aquatic ecosystems. For example, temperate lakes with similar thermal stratification have synchronous chlorophyll *a* concentrations (Baines et al. 2000), variability in flow can synchronize organic matter in streams and rivers (Erlandsson et al. 2008), and increases in nutrients and altered flow regimes can affect the quantity, quality, and timing of basal resources (Kominoski and Rosemond 2012). Marine disturbances like tidal extension and saltwater intrusion are rapidly influencing coastal ecosystem biogeochemistry (Ensign and Noe 2018, Tully et al. 2019). In South Florida, sea levels have rapidly increased in the last decade (Wdowinski et al. 2016), and restoration of the Everglades is recently increasing water levels in many freshwater ecosystems (Dessu et al. 2018). Here, we studied long‐term (2001–2017) monthly variability in water column dissolved organic carbon (DOC), total nitrogen (TN) and phosphorus (TP) concentrations and bacterioplankton productivity in coastal wetlands exposed to multiple disturbance events, as well as long‐term increases in water levels. Data were collected from freshwater‐to‐marine gradients of two subtropical coastal drainages that differ in drainage area, residence time, inundation depth, tidal extent and availability of the limiting nutrient, phosphorus. We asked (1) How do spatial and temporal patterns of variability in key nutrients and DOC compare between freshwater and marine coastal ecosystems in response to different types of disturbances? And (2) how do different disturbance types (e.g., fires, low‐temperature anomalies, droughts, floods, hurricanes) interact with long‐term increases in water levels to influence the magnitude and extent of disturbance legacies that influence carbon (DOC), nutrients (TN, TP), and aquatic microbes (bacterioplankton)? We used time series and spatiotemporal synchrony analyses (i.e., similarity; Baines et al. 2000) of long‐term water temperature, chemistry, and microbial activity datasets from coastal wetlands and estuaries to test these questions. We predicted that disturbance events would increase carbon (DOC) and nutrient concentrations (TN, TP) in both freshwater and marine ecosystems due to mobilization of inorganic and organic matter that would interact with increasing water levels (i.e., higher hydrologic connectivity) to increase spatiotemporal synchrony. We anticipated that hurricanes and droughts would increase and synchronize the limiting nutrient, TP (Castañeda‐Moya et al. 2010, Davis et al. 2018), across an estuary gradient, and subsequently increase and synchronize bacterioplankton productivity. We also anticipated that fires and cold events would increase DOC and TP from enhanced detrital production from plant and animal mortality (Boucek and Rehage 2014, Boucek et al. 2016, Danielson et al. 2017, Davis et al. 2018), and reduced P‐limitation would increase N‐fixation (Schafer and Mack 2010, Butler et al. 2018). Finally, we predicted that stoichiometric responses (DOC:TN, DOC:TP, and TN:TP) would depend upon disturbance type but largely be driven by disproportionate increases in TN and TP relative to DOC.

## Methods

### Site description and experimental design

We analyzed long‐term data from the Florida Everglades, a Ramsar Wetland of International Importance, International Biosphere Reserve, and U.S. Long Term Ecological Research (LTER) site of the Florida Coastal Everglades (FCE) LTER located in Everglades National Park (a United Nations Education, Science, and Cultural Organization World Heritage Site), in South Florida, USA. The Florida Everglades is a vast oligotrophic, P‐limited, subtropical coastal landscape that supports extensive and diverse wetlands with varying hydrology, productivity, and relative nutrient limitation (Noe et al. 2001, Childers et al. 2006, Rivera‐Monroy et al. 2011, Castañeda‐Moya et al. 2013). Although TP concentrations in surface waters are extraordinarily low (range 0.25–0.65 μmol/L) in Florida Bay and the Gulf of Mexico (Fourqurean and Zieman 2002, Boyer 2006), marine inputs of TP are slightly higher in the coastal mangrove area than in the inland freshwater wetlands. As a result, marine disturbances as well as saltwater intrusion coincides with P delivery to interior ecosystems, making the Everglades an ideal location to study climate‐ and hydrologic‐induced changes in biogeochemical cycling in coastal wetlands (Rivera‐Monroy et al. 2019).

**Figure 1:**
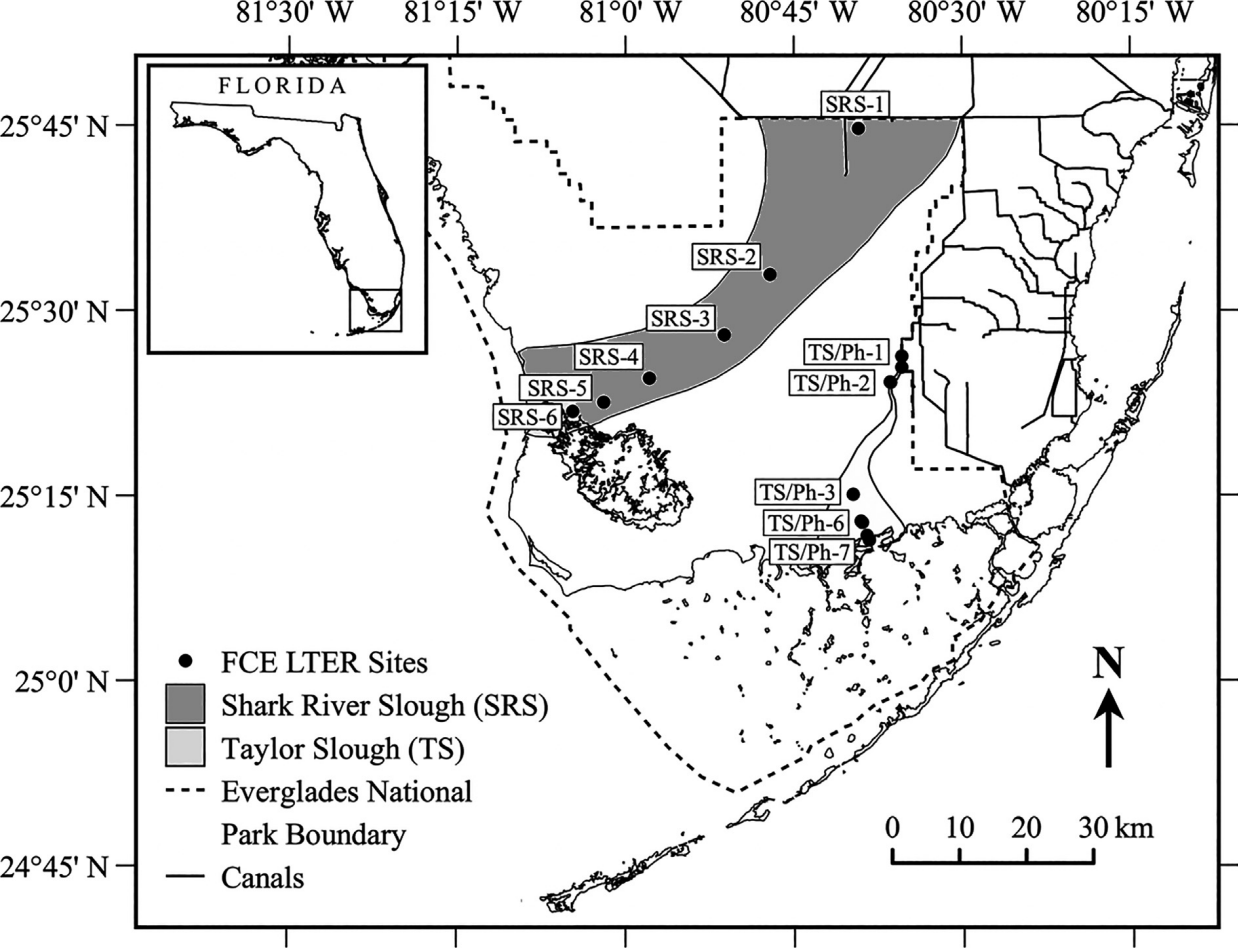
Location of the study sites in the Florida Coastal Everglades (FCE), Everglades National Park (ENP) in South Florida, USA. Sites along Shark River Estuary include long‐hydroperiod freshwater marshes (SRS‐1, ‐2, ‐3) and riverine tidal mangroves (SRS‐4, ‐5, ‐6). Sites along Taylor Slough/Panhandle include short‐hydroperiod marshes (TS/Ph‐1, ‐2, ‐3) and micro‐tidal mangroves (TS/Ph‐6, ‐7). The ecotone coincides with SRS‐4 in SRS and TS/Ph‐3 in TS/Ph. CS is a site on Cape Sable that has a long‐term surface water temperature record for mangroves. All sites are part of the FCE Long‐Term Ecological Research (FCE‐LTER) program.

The FCE‐LTER sites are located along two main hydrologic drainages, Shark River Slough (SRS) and Taylor Slough/Panhandle (TS/Ph; Fig. 1). Shark River Slough is a larger, deeper drainage with oligotrophic, long‐hydroperiod (inundated> 9 month/yr) wetlands dominated by sawgrass and benthic microbial mats (periphyton) that connect to the Gulf of Mexico through highly productive riverine mangrove forests exposed to semi‐diurnal, tidal exchange (mean amplitude is 1.1 m; Provost 1973, Wanless et al. 1994, Chen and Twilley 1999, Childers et al. 2006, Ewe et al. 2006, Castañeda‐Moya et al. 2013). In contrast, TS/Ph is a smaller, shallower drainage containing highly oligotrophic short‐hydroperiod (inundated < 9 month/yr) wetlands, also dominated by sawgrass and thick periphyton mats, that connect to a wind‐driven, micro‐tidal system of short‐stature (scrub) mangrove forests and the expansive, shallow Florida Bay estuary. Productivity along this drainage peaks in the marsh–mangrove “oligohaline” ecotone due to coastal groundwater upwelling (TS/Ph‐3, Fig. 1; Childers et al. 2006, Ewe et al. 2006, Troxler et al. 2014). In TS/Ph, water flow and hydrologic influence from Florida Bay are also determined by seasonal precipitation, upland runoff, and wind (Sutula et al. 2001, Michot et al. 2011). The connectivity to marine and freshwater groundwater sources is an important driver of carbon and nutrient dynamics in both SRS and TS/Ph (Price et al. 2006, Saha et al. 2011, Flower et al. 2016, 2017a,2017b). The SRS drainage primarily accretes peat soils while the TS/Ph drainage is dominated by marl (calcium carbonate) soils precipitated abiotically and by cyanobacteria residing in periphyton mats, in part due to historical drainage of the Everglades (Davis and Ogden 1994). This study focuses on the long‐term water quality dynamics of six FCE‐LTER sites along SRS (three freshwater marshes [SRS‐1, ‐2, ‐3], three riverine mangrove forests [SRS‐4, ‐5, ‐6]) and five sites along TS/Ph (three freshwater marshes [TS/Ph‐1, ‐2, ‐3], two scrub mangroves [TS/Ph‐6, ‐7]; Fig. 1). In general, mangrove wetlands of SRS have higher water TP and lower DOC concentrations than inland freshwater, peat‐based marshes that have higher TN and DOC concentrations (Childers et al. 2006, Regier et al. 2016). Mangrove wetlands of TS/Ph generally have higher water TN, TP, and DOC concentrations than inland freshwater, marl‐based marshes that are low in organic matter and nutrients (Price et al. 2006). Hereafter, we refer to wetland type among our sites as freshwater (marshes) and marine (mangroves).

### Disturbance history

**Figure 2:**
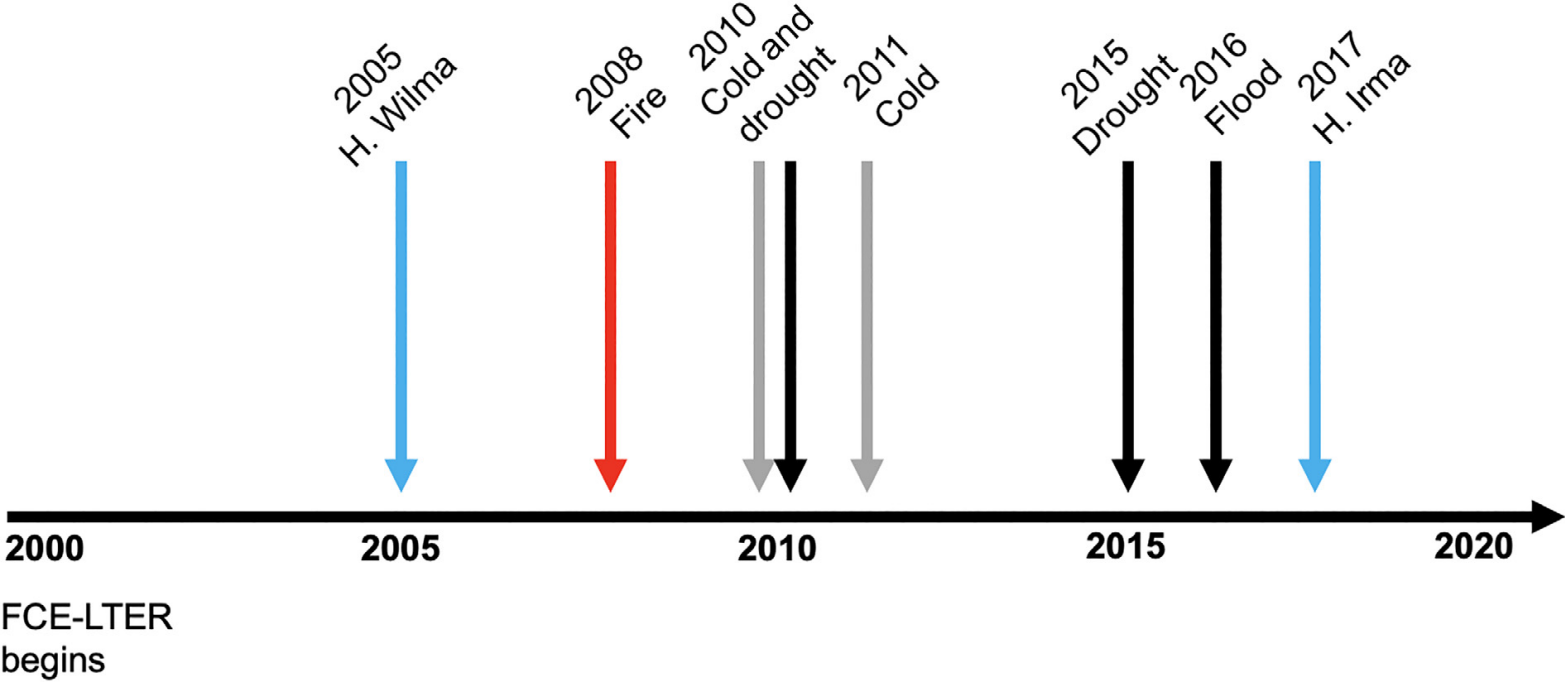
Disturbance history of ecologically important events that occurred along the Shark River Slough and Taylor Slough/Panhandle gradients associated with the studies sites for the Florida Coastal Everglades Long Term Ecological Research program. Disturbances are indicated by black (droughts, flood), red (fire), blue (hurricanes, H.), and grey lines (freeze events).

The FCE and South Florida have experienced multiple ecologically important disturbances within the past two decades (Fig. 2). Two major hurricanes in 2005 (Hurricane Wilma) and 2017 (Hurricane Irma), two cold events (2010, 2011), a landscape fire in 2008, two droughts (2010, 2015), and extensive flooding associated with El Niño in 2015 have occurred in both SRS and TS/Ph drainages (Fig. 2).

### Surface water physicochemistry

Surface water level data were collected from 2000 to 2017 nearby stations from the U.S. Geological Survey Everglades Depth Estimation Network (Shark River Slough [NP201, P36, MO‐215, TE, Gunboat Island, SH3]; Taylor Slough/Panhandle [NTS1, TSB, E146, UTR, TRM]) (USGS/EDEN 2018). Continuous surface water temperature was measured at representative marsh sites (TS/Ph‐1 and SRS‐2) from 2008 to 2017 and at a representative mangrove site (25°09ʹ N, 81°05ʹ W; Lorenz 2018) from 2005 to 2017 using Type‐T Thermocouples (Omega Engineering, Norwalk, Connecticut, USA) or Hydrolab/Hach Quanta Transmitters (Kempten, Germany) stored on data loggers (Campbell Scientific, Logan, Utah, USA). Surface water grab samples were collected from all field sites every 3–4 weeks, placed immediately on ice, and brought to the laboratory for analysis of DOC, TN, and TP. Total P was analyzed following Solorzano and Sharp (1980), and TN was measured with an Antek TN analyzer (Antek Instruments, Houston, Texas, USA). DOC was analyzed on filtered (0.7‐μm GF/F filters; Whatman, Maidstone, UK), samples using a Shimadzu TOC Analyzer (Shimadzu Corporation, Columbia, Maryland, USA). All water quality nutrient (TN, TP) and DOC analyses were conducted at the Southeast Environmental Research Center Nutrient Analysis Laboratory (SERC‐NAL; Gaiser and Childers 2018, Troxler 2018, Troxler and Childers 2018). SERC‐NAL followed strict internal and external QA assurance practices and is NELAC Certified for non‐potable water‐General Chemistry under State Lab ID E76930.

### Bacterioplankton productivity

We analyzed bacterioplankton from monthly surface water grab samples collected from the main channel at all sites from 2001 to 2017 (Briceño 2018). Samples were collected, placed on ice, and filtered (0.2‐μm) with nitrocellulose filters at the laboratory within 24 h. Bacterioplankton productivity was quantified as uptake of tritiated thymidine during 1‐h incubations in the laboratory at 22°C (see Bell 1993).

### Data analyses

We analyzed daily surface water levels in freshwater and marine coastal ecosystems using the Theil‐Sen slope with 95% confidence intervals using the trend package in R. To test how spatial and temporal patterns of biogeochemistry vary between freshwater and marine coastal ecosystems, we analyzed monthly time series of surface water DOC, TN, and TP concentrations, and bacterioplankton productivity along SRS and TS/Ph using separate analysis of covariance (ANCOVA) for both drainages with wetland type (freshwater, marine) as a predictor variable, year as a covariate, and their interaction.

To test for the effect of discrete cold events on water temperature time series among wetland types, we analyzed monthly surface water temperature from 2008 to 2017 for two freshwater marsh sites (TS/Ph‐1, SRS‐2) and from 2005 to 2017 for a mangrove site (CS).

We tested for spatial and temporal similarity, or synchronization, of long‐term DOC, TN, and TP concentrations, and bacterioplankton productivity using the synchrony package in R (Gouhier and Guichard 2014). We computed empirical variograms to quantify temporal and spatial patterns of auto‐ and cross‐correlated variability in all parameters, and assessed their statistical significance using Monte Carlo randomizations and Pearson product–moment correlation (Bjornstad et al. 1999, Fortin and Dale 2005). Spatiotemporal synchrony was calculated as the relative correlation between each variable across a range of sampling distances, or lag distances (the distances between each pair of sampling points plus a tolerance width within each transect) (Gouhier and Guichard 2014). All analyses were conducted using the statistical software R v. 3.3.3 and RStudio v. 1.0.136 (R Core Team 2017).

## Results

### Surface water physicochemistry

**Figure 3:**
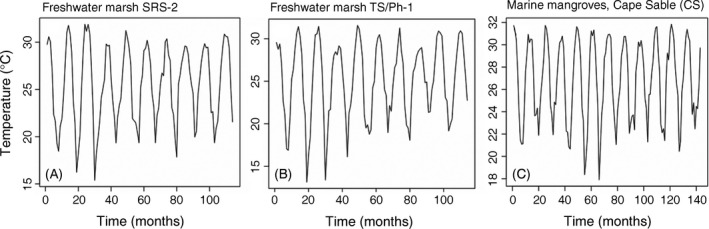
Surface water temperatures from in the Florida Coastal Everglades, Everglades National Park, Florida, USA. Time series of mean monthly surface water temperatures were analyzed from 2008 to 2017 in freshwater marshes of (A) Shark River Slough (SRS‐2) and (B) Taylor Slough/Panhandle (TS/Ph‐1), and from 2005 to 2017 at a representative mangrove site in Cable Sable (C). See legend of Fig. 1 for site names and locations.

Mean monthly water temperatures ranged from 14°C to 32°C at both TS/Ph‐1 and SRS‐2 freshwater marsh sites, and from 18°C to 32°C at the Cape Sable mangrove site (Fig. 3). Declines in water temperatures in 2010 and 2011 associated with severe low‐temperature events were detected at all three sites (Fig. 3A–C).

**Figure 4:**
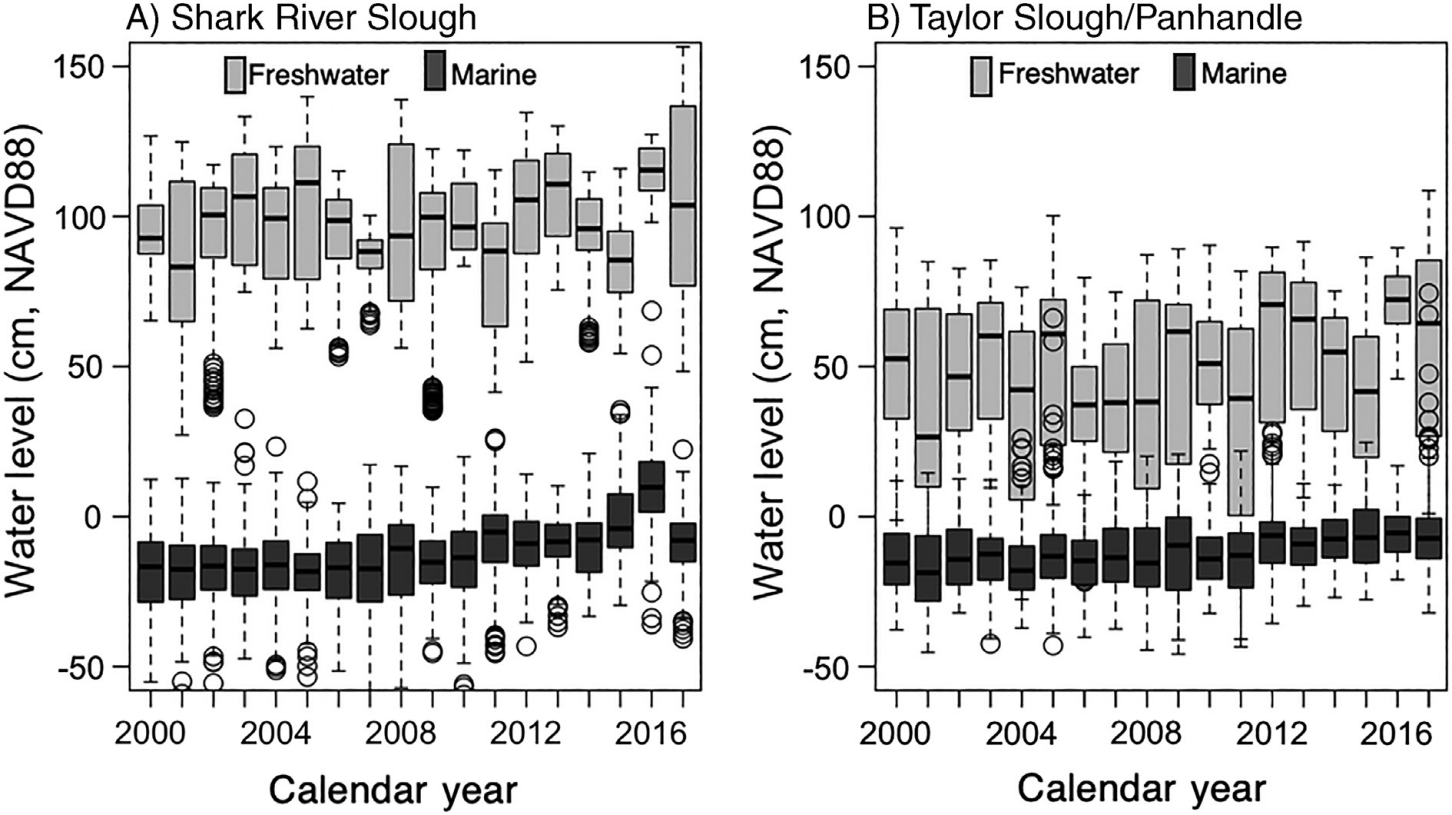
Box and whisker plots of median (black line), upper and lower quartiles (box edges) of annual surface water levels among freshwater (gray) and marine wetlands (dark gray) of (A) Shark River Slough and (B) Taylor Slough Panhandle from 2000 to 2017. Freshwater wetlands refer to marshes, and marine wetlands refer to mangroves. Whiskers represent the maximum and minimum values; open circles represent outlier values.

All surface water levels within freshwater and marine wetlands of SRS and TS/Ph increased over time (Fig. 4). Slopes ranged from 0.002 cm/yr at SRS freshwater and TS/Ph marine sites, 0.003 cm/yr at TS/Ph freshwater sites, and 0.004 cm/yr at SRS marine sites (*P* < 0.001).

**Figure 5:**
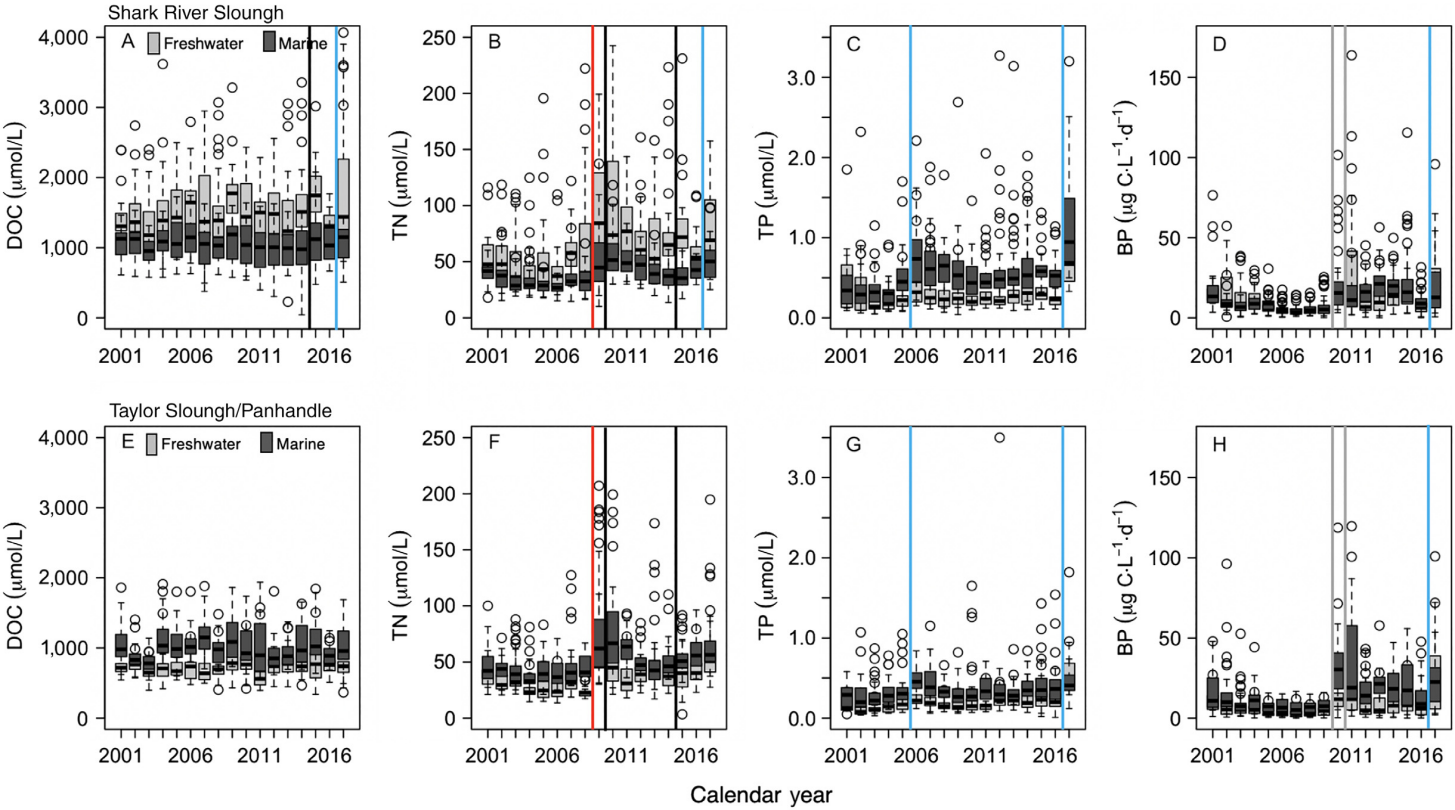
Box and whisker plots of median (black line), upper and lower quartiles (box edges) of annual surface water (A, E) dissolved organic carbon (DOC), (B, F) total nitrogen, (C, G) total phosphorus concentrations, and (D, H) bacterioplankton productivity among freshwater (gray) and marine wetlands (dark gray) of Shark River Slough and Taylor Slough Panhandle from 2001 to 2017. Disturbances are indicated by black (droughts, flood), red (fire), blue (hurricanes), and gray lines (freeze events). Freshwater wetlands refer to marshes, and marine wetlands refer to mangroves. Whiskers represent the maximum and minimum values; open circles represent outlier values.

Concentrations of DOC, TN, and TP in SRS and TS/Ph varied by wetland type (freshwater, marine) and year, except for DOC in TS/Ph that remained similar over time (Table 1). Concentrations of DOC in SRS were higher in freshwater marshes than marine wetlands, and we detected high temporal variability in DOC along SRS (Fig. 5A). Abrupt increases in DOC concentrations in freshwater marshes of SRS were detected following drought and subsequent flooding in 2015 and Hurricane Irma in 2017 (Fig. 5A). In TS/Ph, DOC concentrations were higher in marine than freshwater wetlands but remained similar over time (Table 1, Fig. 5E). Surface water TN concentrations ranged from ~ 10 to >200 µmol/L along SRS and TS/Ph (Fig. 5B, F). Abrupt increases in TN concentrations in both drainages occurred in 2009 following a landscape fire in 2008, and droughts in 2010 and 2015 (Fig. 5B, F). In SRS, TN concentrations also increased following Hurricane Irma in 2017 (Fig. 5B). Surface water TP concentrations ranged from ~0.2 to 3.0 µmol/L along SRS (Fig. 5C) compared to ~0.0 to 1.0 µmol/L along TS/Ph sites (Fig. 5G). Surface water TP concentrations were higher in SRS and TS/Ph marine sites compared to freshwater sites (Fig. 5C, G). Abrupt increases in TP concentrations in 2005 and again in 2017 were detected along SRS and TS/Ph, which coincided with Hurricanes Wilma and Irma, respectively (Fig. 5C, G).

**Table 1:** Analysis of covariation model results (*P* values) of biogeochemical responses testing wetland type (freshwater, marine) as a predictor variable, year as a covariate, and their interaction.

Site and predictor	Dissolved organic carbon	Total nitrogen	Total phosphorus	Bacterioplankton productivity
Shark River Slough				
Year	0.02	<0.001	<0.001	<0.001
Type	0.001	<0.001	<0.001	0.32
Year × type	0.26	<0.001	<0.001	<0.001
Taylor Slough/Panhandle				
Year	0.26	<0.001	<0.001	<0.001
Type	<0.001	<0.001	<0.001	<0.001
Year × type	0.42	0.76	0.43	0.23

**Figure 6:**
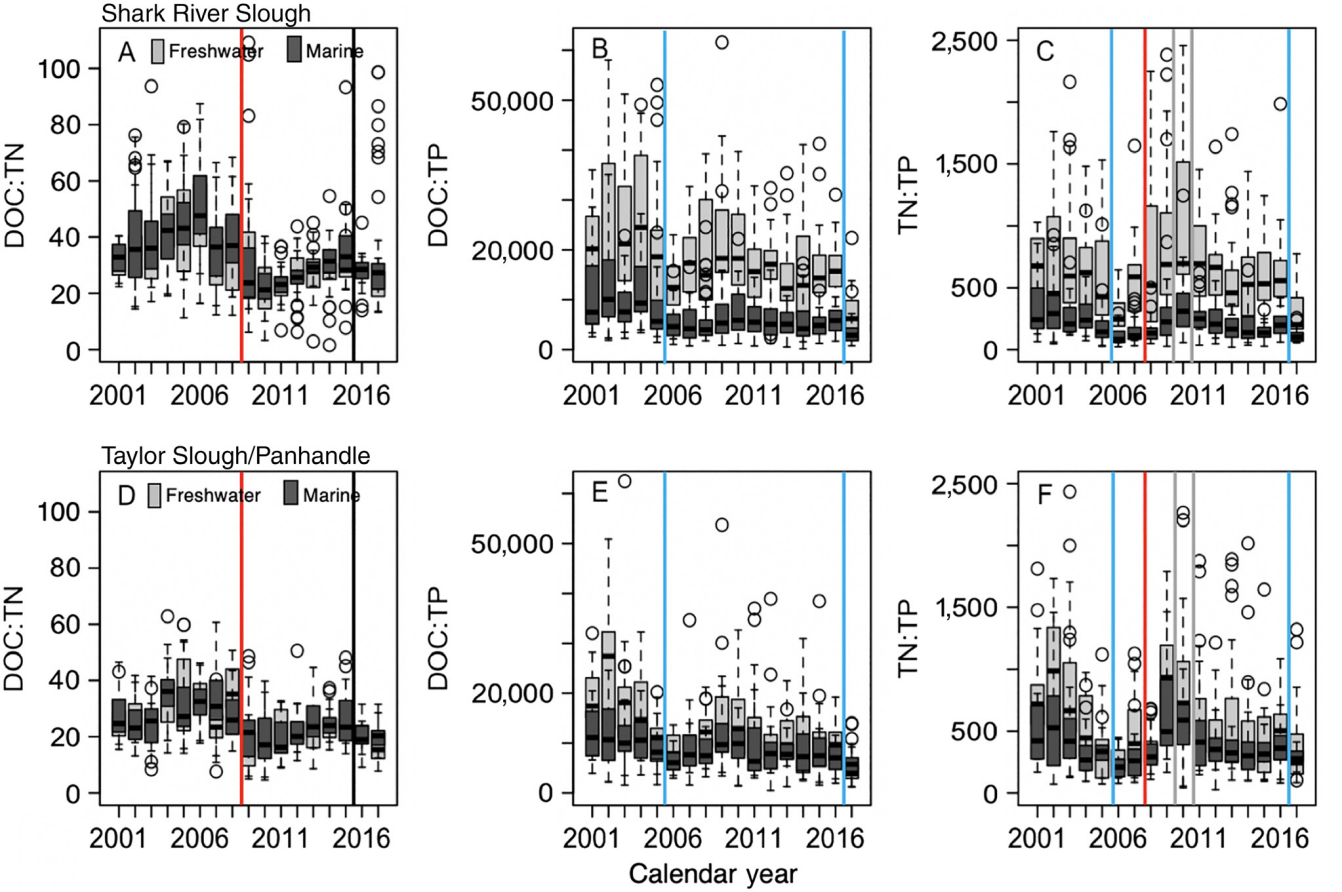
Box and whisker plots of median (black line), upper and lower quartiles (box edges) of annual surface water dissolved organic carbon (DOC), total nitrogen, and total phosphorus molar ratios [(A,C) DOC:TN, (B,D) DOC:TP, (C,F) TN:TP] among freshwater (gray) and marine wetlands (dark gray) of Shark River Slough and Taylor Slough Panhandle from 2001 to 2017. Disturbances are indicated by black (droughts, flood), red (fire), blue (hurricanes), and gray lines (freeze events). Freshwater wetlands refer to marshes, and marine wetlands refer to mangroves. Whiskers represent the maximum and minimum values; open circles represent outlier values.

We detected long‐term changes in dissolved nutrient elemental stoichiometry in response to disturbances. Water DOC:TN were similar among freshwater and marine wetlands in both SRS and TS/Ph and decreased following a landscape fire in 2008 and drought in 2015 (Fig. 6). Water DOC:TP and TN:TP were higher in freshwater than marine wetlands in SRS (P availability marine> freshwater) but similar in TS/Ph, and decreases in DOC:TP and TN:TP in both drainages coincided with pulses of marine and freshwater in response to hurricanes (2005, 2017) and freeze events (2010, 2011; Fig. 6).

### Bacterioplankton productivity

Bacterioplankton productivities in SRS and TS/Ph varied by year and site (Table 1) and increased abruptly in 2010 across all wetlands of SRS (range ~5 μg C·L^−1^·d^−1^ to >20 μg C·L^−1^·d^−1^) and TS/Ph (range ~5 μg C·L^−1^·d^−1^ to >20 and 30 μg C·L^−1^·d^−1^) that coincided with two low‐temperature anomalies that caused documented plant and animal mortality, as well as following Hurricane Irma in 2017 (Fig. 5D, H).

### Spatial and temporal patterns of synchrony

**Figure 7:**
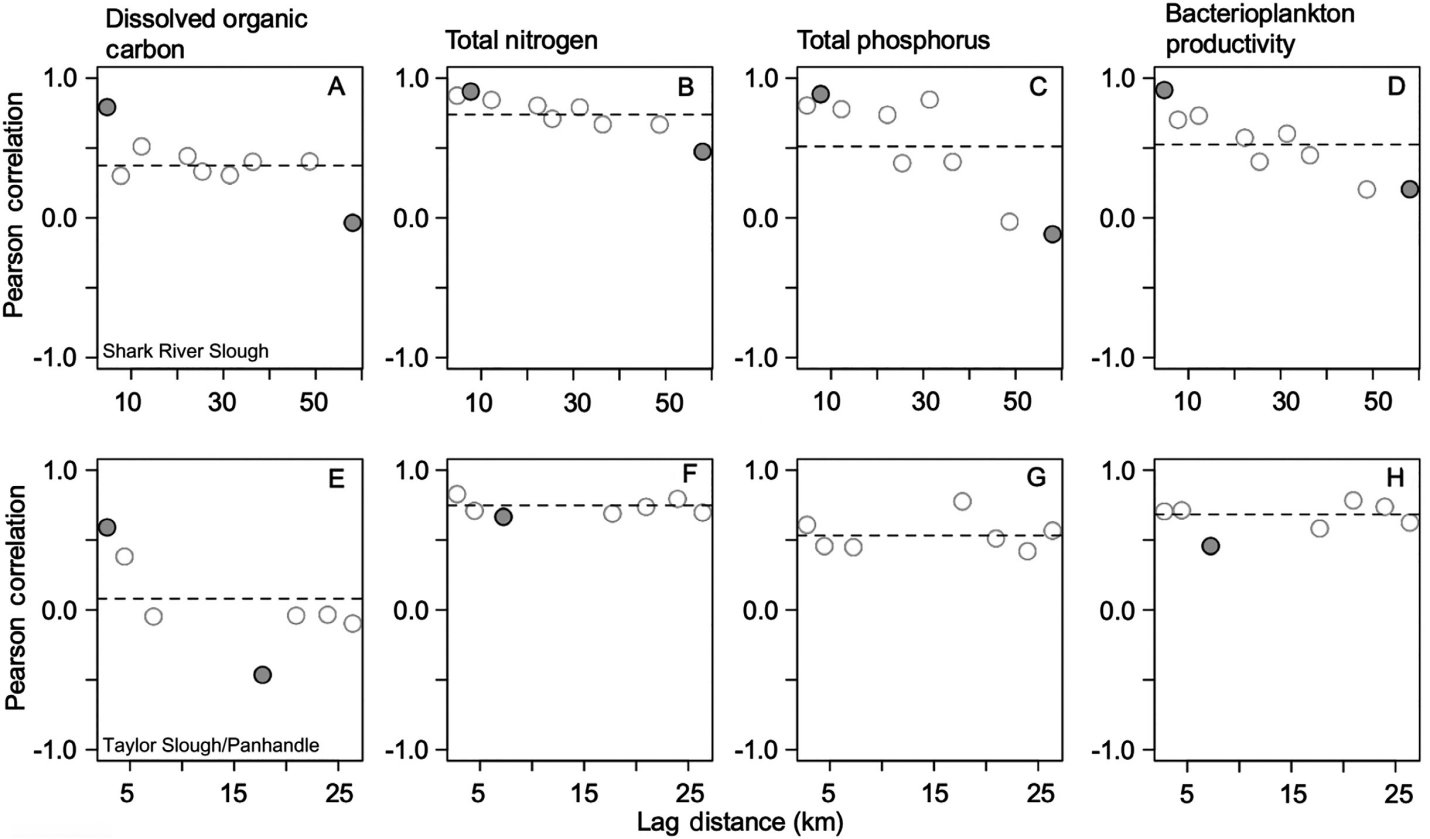
Spatial and temporal synchrony of long‐term mean dissolved organic carbon, total nitrogen, and total phosphorus concentrations, and bacterioplankton productivity along the estuarine‐freshwater gradient of (A–D) Shark River Slough (SRS) and (E–H) Taylor Slough/Panhandle (TS/Ph) from 2001 to 2017. Positive and negative correlations with lag distance indicate relative synchrony or asynchrony, respectively. Horizontal dashes are mean values across spatial gradients of both SRS and TS/Ph transects. Solid circles denote significant (*P* < 0.05) departures from means.

In SRS, surface water DOC, TN, and TP concentrations, and bacterioplankton productivity, were strongly positively correlated with lag distance between sampling locations within each transect (Fig. 7A–D). In TS/Ph, DOC, TN, and bacterioplankton productivity were positively correlated with lag distance, but surface water TP was not correlated with lag distance between replicate sampling locations (Fig. 7E–H).

## Discussion

Disturbance events in coastal ecosystems are increasing alongside long‐term increases in sea level, saltwater intrusion, and associated biogeochemical transformations (Ensign and Noe 2018, Tully et al. 2019). Based on previous theoretical and empirical research that found hydrologic‐ and temperature‐driven disturbances synchronize aquatic ecosystems (Baines et al. 2000, Erlandsson et al. 2008, Kominoski and Rosemond 2012), we predicted that disturbance events would interact with long‐term increases in water levels in both freshwater and marine ecosystems to increase spatiotemporal similarity (i.e., synchrony) of organic matter, nutrients, and microbial activities. We predicted that hurricanes and droughts would increase and synchronize TP (Castañeda‐Moya et al. 2010, Davis et al. 2018), which would increase and synchronize bacterioplankton productivity. We expected DOC and TP increases following fire would reduce P limitation and increase N fixation (Schafer and Mack 2010, Butler et al. 2018). We also predicted DOC and TN increases following cold events, as detrital organic matter likely increases with mortality of plants and animals (Boucek and Rehage 2014, Boucek et al. 2016, Danielson et al. 2017, Davis et al. 2018). Stoichiometric responses (DOC:TN, DOC:TP, and TN:TP) were expected to be driven by increases in TN and TP. We found that different types of disturbances, hurricanes, fires, and low‐temperature anomalies, similarly increased long‐term water nutrient concentrations (TN, TP) for several years to as long as a decade, but not DOC concentrations in either freshwater or marine ecosystems. DOC was derived from upstream freshwater marshes for SRS (Regier et al. 2016; P. Regier, *unpublished data*) and mangrove‐derived for TS/Ph (Fig. 5), and concentrations in DOC didn’t change after any disturbance type. As such, water DOC:TN and DOC:TP ratios decreased for nearly a decade following nutrient pulses from fires and hurricanes in freshwater and marine wetlands of SRS and TS/Ph. In contrast, fires, hurricanes, and low‐temperature events increased freshwater TN:TP ratios for a decade in SRS but only for 2 yr in TS/Ph (Fig. 6). Increases in surface water TP concentrations in marine wetlands of SRS associated with Hurricane Wilma in October 2005 have increased the baseline and variance of P at those sites, as compared to concentrations from 2001 to 2005, and abruptly increased again in 2017 following Hurricane Irma (Fig. 5). Increases in surface water TN concentrations occurred at freshwater sites of SRS and marine and freshwater sites of TS/Ph for 2 yr following large and intense fires (Fig. 5). Bacterioplankton productivities increased throughout freshwater and marine coastal wetlands of both drainages following the 2010 and 2011 low‐temperature anomalies and have remained elevated for nearly a decade (Fig. 5). Legacies of disturbance manifested in different nutrient and microbial responses, suggesting that disturbance interactions can have complex and dynamic effects on wetland biogeochemistry. Our long‐term analyses also indicate that organic carbon in dissolved form may be less sensitive to disturbances than nutrients and microorganisms in coastal aquatic ecosystems, and that any excess DOC released is likely rapidly consumed by heterotrophic bacteria.

### Disturbances and biogeochemical cycles

Understanding the spatial and temporal patterns and drivers of biogeochemical cycling among ecosystems is critical given widespread mobilization of N and P that are linked to disturbance (Carpenter et al. 1998, Elser et al. 2007). In subtropical and tropical ecosystems, hurricanes increase nutrients from both marine (e.g., sediments; Castañeda‐Moya et al. 2010) and freshwater sources (e.g., TP loading; Dessu et al. 2018). In addition, sea‐level rose at rates faster than expected between 2006 and 2013 (Wdowinski et al. 2016). Our long‐term research has revealed that Everglades restoration is also increasing water levels throughout freshwater and marine ecosystems (Fig. 4; Dessu et al. 2018). Abrupt increases in surface water TP concentrations along SRS associated with Hurricane Wilma in October 2005 increased the median and variance of TP at those sites for years to over a decade, compared to concentrations from 2001 to 2005 (Fig. 5C; Davis et al. 2018). Indeed, TP concentrations along marine sites of SRS increased sharply following Wilma in 2005 and maintained those concentrations until 2017 when they abruptly increased again after Hurricane Irma (Fig. 5C). Elevated surface water TP concentrations were also observed throughout TS/Ph (Fig. 5G), where increases in TP appear to be driven by increased water levels from water management and pulsed freshwater deliveries during Hurricane Wilma (Fig. 5G). In addition, disturbances like fire and low‐temperature events release organically bound nutrients (Schafer and Mack 2010, Butler et al. 2018, Davis et al. 2018), which corroborate with findings from this study.

Disturbances and long‐term environmental changes synchronize the timing, sources, and production of organic matter and nutrients in aquatic ecosystems. Hydrologic‐ and temperature‐driven environmental changes and disturbance events can determine the quantity, quality, and phenology of basal resources (e.g., detrital or algal organic matter) and nutrients as well as their use by microbes (Baines et al. 2000, Erlandsson et al. 2008, Kominoski and Rosemond 2012). In this study, we found that low‐temperature disturbance events increased the magnitude and spatial similarity of bacterioplankton productivity. Synchronized increases in bacterioplankton productivity among all sites since 2010 (Figs. 5D, H, 7D, H) suggest that fundamental shifts in microbial activities are disturbance legacies of the cold events (Fig. 3). In addition, our results also suggest that increased P loading from marine and freshwater sources (Dessu et al. 2018) have decreased P‐limitation of bacterioplankton throughout our study sites (Lennon and Pfaff 2005). Observed increases in bacterioplankton productivity appear to have been triggered by the 2010 and 2011 cold snaps, but also coincided with disturbance‐driven increases in TN and TP and a threefold increase in rate of sea‐level rise and water level increases throughout the landscape (Wdowinski et al. 2016, Dessu et al. 2018). Thus, increases and synchrony in bacterioplankton productivity in both freshwater and marine wetlands in our study are likely explained by multiple disturbances interacting with increased water levels. A combination of high‐energy storms and extreme events (Danielson et al. 2017, Davis et al. 2019), and increases in water level and sea‐level rise (Wdowinski et al. 2016, Dessu et al. 2018) have also likely contributed to long‐term patterns of increasing TN and TP concentrations and synchrony in estuarine wetlands. Groundwater salinity and TP concentrations in estuarine and brackish wetlands will likely continue to increase with sea‐level rise under current water management regimes (Flower et al. 2017b).

### Sea‐level rise and saltwater intrusion

Marine disturbances like tidal extension and saltwater intrusion are rapidly influencing coastal ecosystem biogeochemistry (Ensign and Noe 2018, Tully et al. 2019). Although restoration projects are increasing freshwater flows to freshwater and marine wetlands of the Everglades (Arik et al. 2015), sea‐level rise and storm surge continue to increase saltwater intrusion throughout estuarine and brackish wetlands (Saha et al. 2011, Zapata‐Rios and Price 2012, Sandoval et al. 2016). Different regions of the Everglades were considerably drier and wetter than under pre‐drainage conditions, as short‐hydroperiod marshes upstream of canals have longer inundation and long‐hydroperiod marshes downstream of canals have shorter inundation periods (McVoy et al. 2011, Kominoski et al. 2019). Phosphorus concentrations have increased from coast to inland sites over time likely due to saltwater intrusion, and desorption and release of TP (Flower et al. 2017b), as well as of TP from droughts and fires (Davis et al. 2018). This TP increase will most likely be enhanced with increasing saltwater intrusion due to sea‐level rise (Wdowinski et al. 2016, Dessu et al. 2018). Climate‐driven increases in sea level and management increases in freshwater flows have increased water levels throughout the FCE landscape (Dessu et al. 2018). More importantly, the concentrations and components of dissolved organic matter and nutrients in the Everglades are driven by relative differences in water level (freshwater and marine) and salinity (Regier et al. 2016, Dessu et al. 2018). Experimental manipulations of saltwater intrusion consistently reveal decreases in C storage and increases in nutrient export in coastal wetlands (Ardón et al. 2013, 2016, Neubauer 2013, Herbert et al. 2015, 2018, Wilson et al. a, b). Saltwater intrusion will continue to increase salinity, alkalinity, and sulfidation in coastal ecosystems (Tully et al. 2019), and our study illustrates how long‐term environmental changes interact with legacies of disturbance to impact biological and biogeochemical responses.

### Advancing disturbance ecology theory—legacies and synchrony

Understanding the complex set of interactions among disturbances and their biogeochemical consequences is paramount for the long‐term protection, management, and persistence of ecosystems. Ecological theory predicts that ecosystems are maintained and enhanced through regular, pulsed disturbances that release and redistribute organic matter and nutrients (Junk et al. 1989, Odum et al. 1995, Polis et al. 1997, Yang et al. 2008, 2010), but how antecedent conditions influence disturbance responses is complex and often unpredictable. Disturbance legacies and their interactions with other global change drivers can lead to nonlinear ecosystem responses (e.g., Bestelmeyer et al. 2011), and further understanding of how disturbance events may have different consequences depending on prior conditions and legacies is needed (Moorhead 1999, Foster et al. 2003, Johnstone et al. 2016). Our study illustrates how different disturbances interact with increases in water levels from freshwater and marine sources to synchronize organic matter, nutrients, and microbial activities in coastal aquatic ecosystems. Our study helps advance disturbance ecology theory, through the use of long‐term ecological research, to explain how disturbance events interact with long‐term environmental drivers to generate disturbance legacies that cause spatiotemporal synchrony. Whether or not disturbances interact to increase or decrease resources and organic matter is critical to the long‐term trajectories of ecosystems (Odum 1969, Kominoski et al. 2018). Given that many ecosystems are increasingly exposed to multiple stressors, using theoretical expectations of how ecosystems respond to disturbance events, disturbance legacies, and other drivers of environmental change will enhance prediction and enable more effective management of organic matter and nutrients in rapidly changing ecosystems worldwide.
